# Biomarkers of Brain Function and Injury: Biological and Clinical Significance

**DOI:** 10.1155/2015/389023

**Published:** 2015-09-02

**Authors:** Diego Gazzolo, Giovanni Li Volti, Antonio W. D. Gavilanes, Giovanni Scapagnini

**Affiliations:** ^1^Department of Maternal Fetal and Neonatal Medicine, C. Arrigo Children's Hospital, 15100 Alessandria, Italy; ^2^Department of Biomedical and Biotechnological Sciences, University of Catania, 95100 Catania, Italy; ^3^Department of Pediatrics, Neonatology and Child Neurology, University of Maastricht, 6229 HX Maastricht, Netherlands; ^4^Department of Medicine and Health Sciences, University of Molise, 86100 Campobasso, Italy

Over the past decade an increasing interest in the use of biomarkers that are disease-specific to improve the diagnostic, prognostic, and therapeutic approach of different human pathologies has been observed.

Biomarkers have to be measurable in body fluids and able to address unmet clinical needs for the detection of a tissue injury. In particular, biochemical markers of brain damage, both in adult and in paediatric population, have been object of growing interest.

From the 90s onwards, deep evidence about brain plasticity throughout a person's lifespan has been accumulated, in contrast to the previous belief of a nonmutable system. Brain, in effect, is a complex network of different cell subsets that can not only be rewired, but it can also be structurally remodeled. The main consequence of neuroplasticity is the ability of several stimuli, from normal daily experience to damage, or recovery, to modulate the brain activity. The brain compensates for damage by reorganizing and forming new connections between intact neurons. Following a brain injury, in fact, a destructive cascade of biological events continues over hours and days that may worsen the patient's condition. Research studies on markers of brain damage both in humans and in specific animal model might help to understand the underlying mechanisms disrupting this plasticity and giving new insight into several human disease states.

In the current special issue, this topic has been addressed in both experimental and clinical studies, in which biomarkers assessment under different brain damage conditions, affecting central or peripheral nervous system, has been investigated. In detail, neuromarkers currently available have been evaluated (i) in cerebrospinal fluid of healthy adults (L. Hajduková et al.), (ii) in neurodegenerative diseases of adult population such as Alzheimer's disease (L. C. Oliveira-Júnior et al. and K. Weaver et al.), and (iii) in paediatric and adult populations complicated by idiopathic scoliosis, epilepsy, and/or requiring medical/surgical procedures (X. Liu et al., H. W. Lee et al., and D. Tomaszewski), in traumatic brain injury or stroke (A. Mangiola et al. and O. J. Kwon et al.), and in congenital heart diseases surgically treated (A. Varrica et al.). In addition, a space has been given to a novel “omics” science thanks to A. Dessì et al. who reported metabolomics pattern in healthy and intrauterine restricted newborns.

Moving to experimental model the key word* “…perinatal origin of adulthood diseases”* has been investigated in (i) a sheep-based model of perinatal asphyxia (E. Strackx et al.), (ii) a rat model of epilepsy (K. Rijkers et al. and C. Ventura-Mejía and L. Medina-Ceja), (iii) a zebrafish model of regeneration to explore the pattern of genes related to brain plasticity and remodelling (C. Kizil et al.), and finally (iv) mouse brain pattern of neural stem cells biomarkers involved in embryonic and adult neurogenesis (S. Zhang and J. Jiao ).

Last but not least, this special issue also focuses on the need of trustable biomarkers for early diagnosis of brain damage when insult has already occurred and clinical symptoms are at a subclinical stage. Future perspectives will regard the role of novel biomarkers in evaluating positive side-effects of neuroprotective strategies.

*Diego Gazzolo* *Giovanni Li Volti* *Antonio W. D. Gavilanes* *Giovanni Scapagnini*

